# The Medical Aspect of Boswell's "Life of Johnson," with Some Account of the Medical Men Mentioned in That Book

**Published:** 1911-06

**Authors:** Bertram M. H. Rogers

**Affiliations:** Physician to the Bristol Royal Hospital for Sick Children and Women


### THE MEDICAL ASPECT OF BOSWELL'S "LIFE OF JOHNSON," WITH SOME ACCOUNT OF THE MEDICAL MEN MENTIONED IN THAT BOOK.

(*Continued.*)

**The Presidential Address, delivered on October 12th, 1910, at the opening of the Thirty-seventh Session of the Bristol Medico-Chirurgical Society.**

BY

BERTRAM M. H. ROGERS, M.D., B.Ch., B.A. Oxon.,

*Physician to the Bristol Royal Hospital for Sick Children and Women.*

---

I NOW come to the third part of my paper, and propose to give you an account of most of the doctors that are mentioned in the *Life of Johnson.* Johnson took a peculiar pleasure in the company of medical men. This of course is very complimentary to our profession, and he certainly was fortunate in coming across several who were not only skilled physicians, as far as the state of medicine at that time would allow, but were literary men of no mean calibre.

EDMUND HECTOR, the surgeon of Birmingham, was one of Johnson's school-fellows at Lichfield, his earliest friend, and remained so all his life. As young men they both lived for six months with a Mr. Warren, a bookseller, sharing a room. Hector was held by Johnson in great affection, and a correspondence was kept up between them till Johnson's death. He supplied Boswell with many details of Johnson's early life which would otherwise have been lost for ever. He outlived Johnson ten years, and lived to see the publication of Boswell's *Life.*

TOBIAS SMOLLETT'S life is so well known that any lengthy biography of him would be out of place. He was the son of Archibald Smollett, and was born in 1721. When twenty years old he became a ship's surgeon in the Royal Navy, and no doubt the life on board is accurately pictured in his novels.

His fame rests more as a writer than as a member of our profession, and Thackeray describes his work *Humphry Clinker* as "the most laughable story that has ever been written since the goodly art of novel writing began." He died at Leghorn on October 21st, 1771.

Among Johnson's most familiar friends OLIVER GOLDSMITH must take a leading place, for Goldie, as he was affectionately called, was one of the famous Literary Club, of which Johnson was the founder. Though well known, I may perhaps be allowed to spend a few minutes in a short account of this genial but irascible genius. He was born in Ireland in 1729 at Lissoy, which is said to be the original Sweet Auburn, the loveliest village of the plain. Goldsmith did not in his youth display any talents, in fact he was looked on as the dunce of the school, thus confirming Johnson's remark that he was a plant that flowered late. After a few years of idleness at Trinity College, Dublin, he went to Edinburgh, Paris, Germany and Switzerland, finally taking a medical degree at Padua. On his return to London he settled down in Southwark and made some sort of practice. An amusing tale is told of his vanity which is well worth repeating. It was the custom in those days to wear a velvet coat, but Goldsmith not having the means to buy a new one got a second-hand garment. Unfortunately a large patch had to be inserted in front, and was so obvious that to hide it Goldsmith was in the habit of covering it with his hat while visiting his patients. The ludicrous attitude not unnaturally called attention to the very defect that he wished to hide, and resulted in not inconsiderable chaff. In his young days he decided to go to Holland, his intention being to teach English as a means of livelihood, and at the same time to study medicine under Albinus. He appears to have forgotten that a knowledge of Dutch was necessary. He introduces this incident in the *Vicar of Wakefield,* no doubt drawing it from his own experience. Having, however, decided to go he not uncharacteristically booked a passage to Bordeaux. It was a stroke of luck that before the boat sailed he was arrested on suspicion of being a Jacobite, and during the fortnight that he spent in jail the ship sailed and subsequently foundered. However, he in due time reached Holland, and wrote home an amusing account of his experiences.

The practice of medicine was not a lucrative pursuit to him, and he found literature not only more agreeable but better able to supply his wants. The story of the sale of the *Vicar of Wakefield* is too well known to be repeated, and the success of the play *She Stoops to Conquer* was phenomenal. Unfortunately Boswell and Goldsmith did not hit it off, and Boswell appears to have taken malicious delight in telling any little story to Goldsmith's discredit. There is no doubt that he was hasty in temper, vain in person, (though pitted with small-pox), and improvident in habits, but for all that he must have been an extremely attractive and lovable person. Johnson had the highest opinion of his literary abilities, for in writing Goldsmith's epitaph for the monument in Westminster Abbey he used the phrase "Nullum quod tetigit non ornavit."

ROBERT LEVETT, died 1782, was a member of the queer household that Johnson collected together, and possibly no one of them was more strange than this man, who had a practice "in physic amongst the lower people" from Marylebone to Houndsditch. He began life as waiter in a Parisian coffee-house, and making the acquaintance of the surgeons who frequented the place, he took a liking for surgery. His friends had obtained for him permission to attend the lectures under the ablest professors. Why Johnson took him into his house no reason is ever given, but he lived there for thirty-six years, dying a few years before Johnson. His patron had a great regard for him though he said, "Levett is a brutal fellow, but I have good regard for him, for his brutality is in his manners, not in his mind." This comes well from a person who, to put it mildly, was not over refined in his habits. When Levett died Johnson composed a poem on him, and remarked that thus "ended the long life of a very useful and very blameless man."

SAMUEL SWINFEN was Johnson's godfather and father of Mrs. Demoulines, another member of Johnson's household. He is probably the first doctor that Johnson ever consulted for his melancholia, and the result was most unfortunate. Johnson, then quite a young man, wrote his symptoms in Latin, and Swinfen was so struck with the acuteness, research, and eloquence of the letter that he so far forgot the obligation of medical secrecy that he showed the letter to his friends. Johnson was not unnaturally very hurt at this breach of confidence, and never quite forgave Swinfen, though he housed Mrs. Demoulines, Swinfen's daughter, for many years. Swinfen was a graduate of Pembroke College, Oxford, and it is supposed that he interested himself in Johnson going there.

JOHN COAKLEY LETTSOM (1744–1815) the celebrated Quaker doctor, born on the island of Little Vandyke, one of the West Indies, in 1744, came to England at the age of six, and was placed under the care of Mr. Fothergill, the brother of the celebrated London physician. After serving an apprenticeship in Yorkshire, he came to London for two years, and returned to his property in Vandyke. His first act was to free all his slaves, a generous deed which rendered him penniless, but after a few years he saved enough from his practice to be able to return to England. Under the patronage of the Society of Friends he soon began to make his way, and with the further assistance of a wife with ample means he was "in a position to command success." Johnson made his acquaintance at a dinner party at Mr. Dilly's the bookseller. This was rather a momentous dinner party. In the first place, Johnson nearly forgot it, in fact he would have done so if Boswell had not called for him; secondly, Johnson put on a clean shirt, a proceeding of which we are told in his own words he was not too fond; and thirdly, the great John Wilkes was there. Wilkes was one of Johnson's detestations, he was the incarnation of all that was vile and wicked, and Johnson, as a Tory, would have coupled his name with that of the father of lies. Fortunately Wilkes said nothing and did nothing that could offend Johnson, in fact very much the other way, for by carefully attending to Johnson's wants at dinner, "a little of the brown—some fat, sir—a little of the stuffing—some gravy—let me have the pleasure of giving you some butter," and so on, he quite got round Johnson, who delighted in the good things of the table. "Sir, sir, I am obliged to you, sir." Lettsom's memory is kept green by the annual lecture at the College of Physicians of London. He died on November 1st, 1815.

His well-known epigram is worth repeating:—

"Whenever patients come to me
I physics, bleeds and sweats 'em
If after that they choose to die
What 's that to I—I. Lettsom."

I feel that some sort of apology is needed for introducing the name of Dr. RADCLIFFE among my list of doctors, for the only reference to him is in a remark of Johnson's on the travelling fellowships that he had founded in the University of Oxford, one of the many benefactions that he bestowed on the University. Johnson's comment on the fellowships gives a suggestion that has been more energetically followed out of recent years, "that they would be of more value if the study of disease abroad among the native races of the world, rather than the personal intellectual advancement of the fortunate recipient of the fellowship, had been their object." He said, "It is wonderful how little good Radcliffe's travelling fellowships have done. I know nothing that has been imported by them. Yet many additions to our medical knowledge might be got in foreign countries. Inoculation, for instance, has saved more lives than war destroys, and the cures performed by Peruvian bark are innumerable; but it is in vain to send our travelling physicians to France and Italy and Germany, for all that is known there is known here. I 'd send them out of Christendom. I 'd send them among barbarous nations."

Dr. Radcliffe was born in 1650 at Wakefield, of humble parentage, and entered the University of Oxford in 1665. His medical knowledge is said to have been of the smallest, but for all that he rapidly gained a reputation as a physician in Oxford. In 1684 he removed to London, and soon established himself there, partly owing to the disfavour that Lower had acquired from espousing the Whig cause. Royal favour soon shone on him, and William III consulted him; but when

Queen Mary died of small-pox her death was, according to Bishop Burnett, attributed to want of skill and neglect on Radcliffe's part. This is, I believe, an unjust accusation, for the Queen, when Radcliffe was called in, was dying. His remark to the dropsical king calls for repetition only, "I would not have your two legs for your three kingdoms," and not unnaturally lost him his Court favour.

Radcliffe, whatever his medical abilities may have been—and Mead bears witness to his penetration and experience—was a successful man, and much in consequence may be forgiven him. All Oxford men will applaud the noble use he made of his wealth, for the Library, the Infirmary, the Observatory, the small quadrangle in University College and the window in its Chapel, are such additions to the University as no other single person can claim. Little wonder then that the University gave him a splendid funeral and burial after a four weeks' lying-in-state. It is curious that no monument or even stone to mark his grave was put in St. Mary's Church till one hundred and sixteen years after his death, though there was a Radcliffe sermon preached in Commemoration week till 1863, when it was abolished.

Radcliffe, when he became a person of position, attempted to trace his ancestors back to some illustrious persons of the same name, but the Heralds' College interfered, and after the doctor's death admonished the University of Oxford not to erect any escutcheon over or upon his monument. It seems as though the University, to save any dispute, solved the difficulty by erecting no monument at all.

Dr. John Rutty was a Quaker of Dublin. There is no particular interest in this otherwise unknown person except that he kept a record of his own shortcomings, an unusual proceeding, for writers as a rule prefer to perpetuate the failings of others. The examples we have are :—

> "A hypochondriac obnubilation from wind and indigestion."
>
> "An overdose of whisky," followed by the next day's record or sequel—"dull, cross and choleric day."

"A little swinish at dinner."
"Sinfully dogged."

These self-analytical remarks were made in a book called "Spiritual Diary and Soliloquies."

MESSENGER MOUNSEY (1693–1788) was not a favourite of Johnson's, as he "swore and talked bawdy," as well as being a Whig in politics, a friend of Sir Robert Walpole and Lord Chesterfield, and a Unitarian. He appears to have been a very eccentric person, and quite unable to follow the advances that medicine made in those days, for he adhered to the tenets of the school of the celebrated Dutchman Boerhaave, which had been in vogue and were out of date for some years. He was educated at St. Mary's Hall, Cambridge, and practised in Bury St. Edmunds, but left there on his appointment as physician to the Chelsea Hospital. He left his body for dissection, and this Mr. Foster carried out. I believe that the terms of his will were carried out to the letter, and the mangled remains thrown into the Thames.

Mounsey's epitaph, written by himself:—

"Here lie my old bones: my vexation now ends:
I have lived much too long for myself and my friends.
As to churches and churchyards, which men may call holy,
'Tis a rank piece of priest-craft, and founded on folly.
What the next world may be never troubled my pate:
And be what it may, I beseech you, O Fate,
When the bodies of millions rise up in a riot,
To let the old carcase of Mounsey be quiet."

THOMAS LAWRENCE (1711–83) entered Trinity College, Oxford, in 1727, and subsequently went to St. Thomas's Hospital. On the resignation of Dr. Nicholls he was appointed Lee Reader in Anatomy in the University of Oxford. Later he migrated to London, and became President of the College of Physicians, which post he held for seven years. Johnson was among his patients, and was, evidently undeterred by his previous experience with Dr. Swinfen, in the habit of writing his symptons in Latin to him, clearly a habit we must not encourage among our patients. Munk, in his book on the College of

Physicians, says: "Few men have been more respected by the College, none probably by their attainments were better qualified for practice, than Dr. Lawrence. Yet as a physician he made but little progress. He was an elegant classical scholar, a good anatomist, and a sound practitioner, but in his endeavours to attain eminence it was his misfortune to fail." It seems that his personal defects and habits stood in the way, for he had "a vacuity of countenance very unfavourable to an opinion of his learning or sagacity, and certain convulsive motions of his head and shoulders gave pain to the beholders, and drew off attention from all that he said."

In the latter part of his life he became very deaf, and Johnson declared, "I have lost the conversation of a learned, intelligent and communicative companion, and a friend whom long familiarity has much endeared. Lawrence is one of the best men whom I have known."

He died in 1783 at Canterbury, where he had retired a year previously, having become paralysed. An inscription of thirty lines, detailing his excellencies, was engraved on a tablet in the Cathedral.

William Heberden (1710–1801) was one of the physicians who attended Johnson in his last illness. He was a Londoner by birth, and was educated at St. John's College, Cambridge, in which town he began practice, at the same time lecturing on Materia Medica. In 1745 he was a candidate at the College of Physicians, and became a Fellow in the following year. Heberden was probably one of the most advanced physicians of the time, recognising what was good in the old, and adopting the newer methods that were coming into practice. As an instance I may mention his treatise *Antitheriaca,* in which he attacked the exceedingly complex mess of drugs that had come into vogue, or which it had gradually become the practice to employ. The particular one that his sally was made against was that known as "Theriaca," the formula of which had been handed down from the Middle Ages on a recipe said to have been composed by Mithridates, King of Pontus, as a specific against all forms of poison. The original prescription contained thirty-eight ingredients, which in the course of ages had grown to fifty-three, and finally reached seventy-five. He was one of the first to encourage clinical medicine, making voluminous notes of his cases both at the bedside and on his return home. Moore says that the publication of these notes after Heberden's death was of the greatest value, and the reading of them even now is illuminating. Though one of the leading men of his time and an eminent Fellow of the College, he never became President; but whether this was due to his modesty or not I cannot tell. He, however, refused the post of physician to George III. From the accounts that exist of him he appears to have been a person of great personal charm, and his works were so highly thought of that they were translated into German, then as now a rare honour.

WILLIAM CULLEN (1712–90) was born of humble parentage in Lanarkshire, and in early life was a barber. After being an apothecary he studied medicine, acted as a ship's surgeon, and began practice in Scholes, and later in Hamilton. Here he became acquainted with William Hunter, and the two practised in partnership. In order to improve their medical knowledge they agreed that one should carry on the practice while the other went to Edinburgh for the winter session. Cullen was in 1746 elected Professor of Chemistry in Glasgow College, and a few years later Professor of Medicine. In 1756 he returned to chemistry, and was professor at Edinburgh, and then again took to medicine, being made professor at the same University. I have already mentioned Cullen when speaking about the various systems of medicine, and most probably he was a man in advance of his times. As an author his books were in great request, and thought so highly of that they were translated into several foreign languages. He was a man of much charity, and supported Burns's widow, and published his poems at his own cost. He was one of the doctors before whom Johnson asked Boswell to lay his case.

WILLIAM CRUIKSHANK (1745–1800) was the surgeon whom Johnson called in with Pott to treat him when he suffered from sarcocele. Johnson recommended him for the post of

Professor of Anatomy at the Royal Academy, vacant by the death of William Hunter. Cruikshank is said to have been the discoverer of urea.

John Armstrong (1709–79) was the author of a book, *The Art of Preserving Health.* He was a Scotchman, and a friend of Thomson the poet. He gives one of the earliest descriptions of enteric fever, which he treated with copious bleeding and stimulants. He was a poet of no mean order, though I do not expect his effusions are much read now.

George Cheyne (1671–1743), before mentioned as the author of the book *The English Malady,* was a Scotch M.D., and is not mentioned in Munk's Roll. He was born in Aberdeen, and migrated to London in 1702. Johnson recommended the book to Boswell, who had complained of fits of depression, and added the shrewd remark, "Do not let him teach you a foolish notion, that melancholia is a proof of acuteness."

Sir Edward Barry, Bart. The only reference to this distinguished Irish physician occurs in a conversation between Johnson and Boswell. Barry had brought out a theory that pulsation caused death by attrition, and that the way to prolong life was therefore to retard pulsation. Johnson was scornful of this suggestion, arguing that as pulsation was strongest in infants, it cannot be the cause of destruction. Barry was a Dublin physician, and was created an M.D. of Oxford, and admitted a candidate of the College of Physicians of London. He was Physician-General to the forces in Ireland, and Professor of Physic in the University of Dublin.

Sir Charles Blagden (1748–1820), was a physician and Secretary to the Royal Society. Munk does not mention him, so he was not a member of the College of Physicians. Johnson describes him as a delightful fellow.

Sir William Butter (1726–1805) was one of the physicians who attended Johnson in his last illness, and was left a legacy by his patient. He came from the Orkneys, and was an M.D. of Edinburgh. Johnson and Boswell met him at Derby, where he was in practice before he came to London. They took tea with him at his house, and visited the china works. He, like the other doctors who looked after Johnson, took no fees from him.

RICHARD WARREN (1731—97) was another of Johnson's medical advisers, but in his case no mention is made in the will of a bequest. He was the son of Archdeacon Richard Warren, and was educated at Bury St. Edmunds and Jesus College, Cambridge. He was much in favour at Court as physician to the Princess Amelia. He was one of the first to break through the formalities that were used among the physicians of that time, formalities which rendered medicine the favourite theme of ridicule with the wits who happened to enjoy good health.

There is one little anecdote connected with our profession which must be told, for it illustrates the extreme touchiness of the medical men of those days over their titles. This sensitiveness has not quite disappeared, and few of us would bring an action at law, with the usual worry and expense, if someone styled us Doctor of Medicine rather than Physician. This was the reason of Dr. MENIS's action against the Royal Infirmary of Aberdeen, for in a translation of the Charter of that Institution from Latin to English, the same phrase in the original was in one place rendered "Physician," while in another, when referring to Dr. Menis, was translated "Doctor of Medicine." The aggrieved party could not get the translation altered, so sued the managers of the Infirmary for damages on account of the supposed injury, holding that the designation was an inferior one, and suggesting that he was not a physician. Boswell's father, one of the Scotch judges, had dismissed the case as groundless, and Boswell had been retained by the managers of the Infirmary as their advocate. The finding did not please Menis, and he appealed for a re-trial. Boswell wrote to Johnson to ask his aid, and no doubt the opinion of the profession in England. Johnson wrote to Lawrence, at that time President of the College of Physicians, whose opinion was that the title of "Doctor of Medicine (or Physic) was the highest that a practiser of physic could have, and implied that he was a physician and a teacher of physic, and that every doctor was legally a physician." Dr. Menis lost his case on the appeal, and had to pay £40 costs. Boswell's comments are curious for an advocate for the respondent, for he argues in Menis's favour, and holds that the court judged wrong, the respondent being in *malafide* to persist in naming Menis in a manner he disliked. It seems that the question of damage that the offence had done his practice or his reputation was not discussed, though this was the real point upon which he based his action.

Walter Shebbeare (1709–88) is described as a Bristol doctor, though as far as I can discover his only connection with this city is that he wrote a book on the Hotwell, then rapidly becoming popular as a cure for diabetes and consumption. If he was one of us we have no great cause to be proud of him, though perhaps his faults lay in belonging to the unpopular political party. He certainly suffered for his opinions, as he was fined, imprisoned, and put in the pillory for a political libel, called *Sixth Letter to the People of England*, 1758. This pillorying was in his case not such a serious affair as was usually so, for owing to some friendliness on the part of the sheriff he was allowed to stand on, instead of in, the pillory, as well as to enjoy other indulgencies. The fact that he was a strong Tory recommended him to Johnson, who did not mind his name being coupled with Shebbeare's. His faults seem to have been overlooked by a benevolent Government—perhaps the Tories were in power—for he received a pension and defended George III 's American policy, one which I may remind you lost us what are now the United States.

Richard Green, of Lichfield, was a relative of Johnson and he attended Lucy Porter, Johnson's stepdaughter. He was an apothecary, and made a museum at Lichfield full of all sorts of oddities and a few valuables. Some of the armour from it is now in the Tower. Johnson entrusted him with the duty of placing an epitaph on the tombstone of his father and mother in St. Michael's Church, Lichfield.

Richard Brocklesbury (1722–97) was the son of an Irishman of Cork, but was born at Minehead. He studied medicine in Edinburgh and Leyden, of which latter University he was an M.D., though subsequently he was created M.D. of

Cambridge and Dublin. In the College of Physicians he held most of the posts, but did not become President. In 1758 he was appointed physician to the army, and served for some time in Germany in the Seven Years' War, and later he was appointed physician to the hospital for the British forces.

He was Johnson's regular doctor, and attended him in his last illness, with several others. He appears to have been a man of a most generous nature, and on one occasion lent Burke, who was in low water, £1,000 which he had bequeathed to him in his will, and he offered to give Johnson £100 a year for life when the Lord-Chancellor, Lord Thurloe, had refused to increase Johnson's pension.

BENJAMIN HOADLEY'S (1705–57) chief claim to be recorded in this paper is that he referred to Johnson as the "Puffy Pensioner." He was the son of a well-known bishop of the same name, and was educated at Corpus Christi College, Cambridge. He was for many years Physician to St. George's Hospital, and in 1742 to the King's Household.

RICHARD MEAD (1673–1754) was the son of a Nonconformist divine, the Rev. Matthew Mead. His education was mostly received abroad at Utrecht and Leyden, at which latter place he was a fellow-student of Boerhaave. Later he travelled in Italy and graduated at Padua, the English Universities being of course closed to Nonconformists. He started practice in Stepney, then a more salubrious neighbourhood than at present, and a book published by him, *The Mechanical Account of Poisons*, brought him fame. He was appointed Physician to St. Thomas's Hospital, and on the death of Dr. Radcliffe moved to his house in Bloomsbury Square, succeeding to much of his practice. Mead was the Prince of Medicine as far as success is concerned, for no man before or since has ever reached the position he held. Johnson said of him that he "lived more in the broad sunshine of life than almost any man." Apart from his profession he was a great collector of books, statuary, coins, gems, prints and drawings, his house was open to all, his purse free to everyone, for he held that what he received from the public he ought to bestow for their advancement.

Perhaps his most worthy act was to persuade the miser bookseller, Guy, to found the hospital known now and for all time as Guy's.

THOMAS NANNINGHAM, Kt., and son of a knight of the same name, was an M.D. of St. Andrew's, who practised in Jermyn Street, and later at Bath. Johnson met him at Keddlestone in 1777. He, with Smellie and William Hunter, was one of the first physicians to practise midwifery, which had till then been mostly in the hands of ignorant midwives, and to raise that branch of our profession to a dignified position. Nanningham established a lying-in ward in the St. James' Parochial Infirmary, Westminster, in 1739, that being the first of its kind in the United Kingdom.

I regret that I am unable to find much information about DR. MARTIN WARD, of Oxford. He was the son of a Dr. Ward, of Worcester, one, if not the sole, founder of the Worcester China Works, and was educated at Winchester and New College, Oxford. His medical training he had at St. Bartholomew's and Edinburgh. He practised in Oxford, and after being Reader—I presume Lee—in Chemistry, he was appointed Lichfield Professor of Clinical Medicine. He died in 1824. Dr. Johnson had " a peculiar pleasure in the company of physicians, which was not abated by the conversation of this learned and pleasing gentleman." It was to Dr. Ward that Johnson made the remark about the Radcliffe Travelling Fellows.

SIR JOHN HILL (1716–75) is said to have been a quack, but I cannot get much information about him. George III asked Johnson his opinion of him, and the criticism appears not to have been favourable.

ROBERT JAMES (1703–76) was born at Kinveston in Staffordshire, was a schoolfellow of Johnson's, and always a friend. He seems to have divided his medical attentions between Sheffield, Lichfield, Birmingham and London. He was at St. John's College, Oxford, but was an M.D. of Cambridge by royal mandate. His chief claim to fame lies in his celebrated fever powders, and in his book on the history of drugs, in the compilation of which it is said that Johnson gave a hand.

Johnson was much attached to him, and had a high opinion of his powers: "No man brings more mind to his profession." Munk says that he tarnished the fair name he might otherwise have obtained by patenting his powders and falsifying their specifications. His dissertation on fevers ran to eight editions.

I am unable to find who NUGENT was, but there was a friend of Johnson's of that name, an original member of the Literary Club. When on the tour to the Hebrides with Boswell they amused themselves one day by making appointments to an imaginary University of Skye, and Nugent was selected to teach Physic. There is in Munk a Christopher of that name who died in 1775.

ALEXANDER DICK, Kt. (1703–85), was one of the leading physicians of Edinburgh, and was invited by Boswell to meet Johnson at dinner in that city just before the tour to the Hebrides. They also dined with him on their return. It was no doubt the friendship thus formed that induced Johnson to ask Boswell to lay his case before Sir Alexander when his last illness was upon him. In return, as we have seen, the packet of rhubarb which Dick had grown in his own garden was sent. Sir Alexander had previously been presented with a gold medal for the best specimen of this drug by the Society of London for the Encouragement of Arts and Manufactures and Commerce. He was at this time eighty-one years old, and had served as President of the College of Physicians of Edinburgh.

DR. GILLESPIE was another of the Edinburgh physicians whom Boswell consulted on Johnson's case, and from this one Johnson received "an excellent *consilium medicum*, all solid practical experimental knowledge." Boswell's father thought so highly of Dr. Gillespie that he settled £200 for five years and £50 during his (Boswell Senior's) life on him as an honorarium to secure his particular attendance.

GEORGE FORDYCE (1736–1802) was one of the original members of the Literary Club. He was an M.D. of Edinburgh, and for some time before he migrated to London lectured on Materia Medica in that University. He became a Physician to St. Thomas's Hospital, and with John Hunter founded the

Society for the Improvement of Medical and Chirurgical Knowledge and the London Medical Lyceum.

JOSEPH NATHANIEL FORD was brother to Johnson's mother. Perhaps he is more celebrated as the father of Parson Ford, "licentious parson," whose ghost was supposed to have been seen at the Hummums—a place where people got themselves cupped. Johnson's wife went to inquire about it, and satisfied herself of the truth of the story. Johnson's belief was that the man who saw it was suffering from a fever at the time, and imagined it.

MARK AKENSIDE (1721–70) is known better as a poet than as a doctor. He was the son of a butcher of Newcastle, studied medicine at Edinburgh, but was not successful in practice in London, though Physician to St. Thomas's and Harveian Orator. He received some injury in youth that crippled him. Johnson had a high opinion of him as a poet, and included him in his *Lives of the Poets*. One sentence may be quoted, "A physician in a great city seems to be the plaything of fortune, his degree of reputation is for the most part casual, they that employ him know not his excellencies, they that reject him know not his deficiencies.

Another of the doctors of Edinburgh to whom Johnson had his case put was ALEXANDER MUNRO. As you are all aware, there were three generations of this name who at different times held the post of Professor of Anatomy in that city. The one that Johnson must have consulted was the second of that name, a greater light in the anatomical world than his father. I believe he is the man after whom the foramen is named. He succeeded his father at the age of twenty-one, and lectured for fifty years. The three Munros lectured continuously for one hundred and twenty-six years.

SAMUEL MUSGRAVE (1732–80) was an M.D. of Leyden, and was born at Washfield in Devon. He was entered at Christ Church, Oxford, and obtained a Radcliffe Travelling Fellowship, and spent several years on the Continent, in Holland and France. On his return he settled in Exeter, but moved later to Plymouth. He got mixed up in some political questions which are too long and too intricate to go into here, but made some statements that he was not able to substantiate and hence got into bad odour. He removed to London, but was not successful as a practitioner, and lived chiefly by writing. He is said to have been an extremely good Greek scholar, but when he read a poem of his composition to Johnson he had to suffer an unfavourable criticism.

JOHN TURTON (1736–1806) was another Radcliffe Travelling Fellow, and on commencing practice was very successful, holding several Court appointments. It was he who attended Goldsmith when dying, and said to him, "Your pulse is in greater disorder than it should be from the degree of fever which you have. Is your mind at ease ?" Goldsmith's answer was, "No, it is not."

When Johnson was threatened with a surgical operation it was PERCIVAL POTT who was called in for consultation, and I have told how his services were after all not required. Pott was born in 1713 in London, and at an early age expressed his wish to become a surgeon. He was apprenticed to a Mr. Nourse, one of the surgeons of Bart's., and was there employed in getting ready the subjects for his chief's lectures on anatomy, as well as in attending at the hospital. The condition of surgery at that time was very deplorable, "the art was miserable, the instruments were clumsy and unmanageable, the operations unscientific, and unnecessarily painful, the established mode of practice, encumbered with a farrago of useless medicines and applications, tended rather to mislead than direct the inquirer, prescriptions too frequently held the place of reason, and want of real knowledge was concealed under a pompous garb and specious demeanour." This is the picture that surgery then presented when Pott began work. In 1736 he, having finished his apprenticeship, took a house in Fenchurch Street, and eight years later he was appointed surgeon to Bart's., and he at once began to remedy some of the old cruel and useless methods. I cannot omit the story, though possibly well known to many of you, of how he came to study the particular fracture that is still known by his name. One day in January, 1756, while riding in Kent Street, Southwark, he was thrown from his horse and had his leg fractured, the bone coming through the skin. Knowing the dangers of these injuries, he refused to be moved, and sent to Westminster for two chairmen to bring their poles. While waiting he purchased a door, and when the men came he had the poles nailed to the door, and himself placed on the improvised stretcher. In this way he was carried to Watling Street, where he then lived, and on a consultation it was decided to amputate his leg. Fortunately his old chief came in at the opportune moment and dissuaded the surgeons from this, and the leg was saved. During the time that Pott was necessarily laid up he discovered that he could write, and it is said that the first accurate account of the fracture known by his name was worked out while he was in bed. He resigned his post at Bart's. in 1787, and died the following year from pneumonia. His last words were, " My lamp is almost extinguished, I hope that it has burned for the benefit of others."

Hermann Boerhaave (1668–1738) was the most famous physician of the eighteenth century. He was the son of a Dutch pastor of Voorhout, near Leyden, at which latter place he began to practise in 1693. He was a man of extraordinary powers, but the part of his career that most interests us is the power he had of clinical instruction. He was a most voluminous writer, and enjoyed a more than European reputation. It is said that a letter sent from China addressed " to the most famous physician of Europe " reached him. He died in 1738 of gout. It is impossible in a paper such as this to give any account of the theories of medicine that Boerhaave introduced, but they may be summed up as follows : Disease was the condition in which the bodily actions were disturbed or unsettled, and take place only with difficulty ; fever was an effort to ward off death. He was the first to use the clinical thermometer, and the lens for the examination of the eye, as well as the first to permanently establish clinical teaching.

The University of Leyden owes a great deal to Boerhaave, for it was in great measure to his reputation that the medical school became so popular. Goldsmith, who was there some years later did not think that medicine was so well taught as in Edinburgh, and the absence of English students he ascribes to the dearness of necessaries and the laziness of the professors.

Sir John Floyer (1649–1734) was a relative of Johnson's mother, and it was on his recommendation that the infant Johnson was taken to Queen Anne to be touched for the King's Evil. He practised at Lichfield, and appears to have been a man of some repute. His chief work lay in observations on the pulse and its relation to the respiration. He also wrote on the use of hot, cold, and temperate baths in England, and ascribed the prevalence of rickets to the custom of only sprinkling infants in baptism and not plunging them into water. He also wrote a book on asthma, which it appears Johnson had read, for he says that Floyer, himself a sufferer from that disease, "panted on to 90," so that he (Johnson) concluded that his, though "constitutional and incurable," and not laying "close siege on life," would not shorten his days. In a later letter he says that Floyer's asthma was not of the same kind as his own, but the book is obscure by want of order.

Giovanni Baptiste Morgagni was one of the numerous Italian physicians who shine so prominently at this period of history. He was born at Forti in 1682, and took his M.D. at Bologna at the age of 16, being assistant to Valsalva. For a very great number of years he was Professor of Anatomy at Padua, and did much to raise the school of medicine there to the pitch of eminence that it reached in the eighteenth century. He was also distinguished as a literary man and an antiquarian. He died in 1771.

When Johnson and Boswell visited this city in 1776 they went to see St. Mary Redcliffe and the room where Chatterton alleged that he found the Rowley manuscripts. This was after the death of Chatterton (1770) and the exposure of the fictitious character of his poems, or rather of the source of his tales. The part that interests us is that the two visitors went to call on Barrett, the Bristol surgeon, who it is always said was completely taken in by the youth, and put in his *History of*

*Bristol* some of the tales. Quite recently a book has been published on Chatterton by Mr. Ingram, who takes an entirely new view of the part that Barrett played in the tragic comedy. Mr. Ingram's view is that Barrett was not the dupe that every-one has always taken him to be, but was, if not the instigator, at least a willing accomplice. He alleges that Barrett connived with his young friend in connection with the famous "De Bergham" pedigree to the extent of translating the Latin epitaphs and the old French mottoes which Chatterton in-corporated in it, and that he tampered with the spelling of the Rowley "transcripts" in order to give them a more archaic appearance. He looks on Barrett as an unscrupulous man of the world who exploited the boy for his own purposes, and in various ways exercised a profoundly evil influence on his mind. I need hardly say that this is an aspect that not only has never been held before, but is one that will have to be proved more abundantly before it is accepted by the literary world, and perhaps especially by the Bristol admirers of the marvellous boy and the misguided surgeon.

Barrett, apart from his connection with Chatterton, was not a person of much importance, his chief claim to fame being his history of this city. How the value of it is depreciated by the incorporation of Chatterton's tales as facts need not be related by me. One can hardly believe that he wilfully concocted a false history of Bristol, as the truth could so easily be proved.

The story of Barrett's connection with the Chatterton manuscripts, as told by Latimer, our most reliable historian, is that the papers were taken to Catcott by Chatterton, and by the former shown to Barrett, who was collecting the materials for his *History of Bristol*, and in this way Chatterton was intro-duced to Barrett. Chatterton at once saw that Barrett was ready to accept anything that would suit his ideas, being quite taken in by him, and after that the supply of fictitious stories more than equalled the demand. How the spuriousness of the documents was recognised by Horace Walpole after conference with Gray and Mason is well known to all, but Chatterton had many followers or rather believers, amongst whom may be counted the President of the Society of Antiquaries. The genuineness of the papers is not now believed in, but it is admitted by everyone that they show a wonderful power and cleverness.

If Ingram's story is true, why, I should like to ask, did not Chatterton include Barrett in his list of persons, more particularly Bristol ones, that he abuses for their neglect of him? Had Barrett been the instigator or even willing accomplice of Chatterton in his imaginary stories, that led to his undoing, why is he alone spared?

In his *Lives of the Poets* Johnson includes SIR RICHARD BLACKMORE, a member of our profession, and one whose poems have long since received the due reward of their merits. For us the chief interest lies in the part that he took in opposition to Sir Samuel Garth in the great fight between the apothecaries and the College of Physicians. In this controversy feeling ran very high, and the most scurrilous poems, pamphlets and libels were published, many of them too coarse to be repeated in these more refined days. The history of the affair is briefly as follows. The apothecaries had been originally mere grocers, and sold drugs as well as the necessary commodities of the kitchen; but they dispensed the prescriptions of the physicians, and so, not unlike the chemist of to-day, assumed a virtue to which they were not entitled. They, although not allowed to do so, presumed to administer drugs on their own responsibility or judgment, nor could they do any operation except bleeding for pleurisy. In James I's reign they obtained a charter and were placed under the control of the College of Physicians, who had the power to inspect drugs and, if found unsatisfactory, confiscate them. As time went on the apothecaries obtained such a position that they defied the College and began to prescribe for themselves. Finally the quarrel broke out over the dispensing of drugs for the dispensaries for the poor, which the junior members of the College ran for their own advancement, and ended in the physicians starting dispensaries and selling the drugs at cost price. This caused a split in the College itself, the opposition refusing to meet the others in consultation. Blackmore in this way came into collision with Sir Samuel Garth. Garth's poem, called the "Dispensary," was a satire on the apothecaries and those who opposed them.

Blackmore was educated at Westminster School and St. Edmund's Hall, Oxford. His siding with the revolutionary party brought him to William III's notice, and resulted in his being made Physician in Ordinary. I doubt whether anyone now reads his poems.

The last two on my list are, though members of our profession, no particular credit to it, but they are two of the numerous irregular practitioners who were allowed to descend to any low practice in order to humbug the public.

To define "quackery" is difficult. If to "talk noisily and ostentatiously," as one dictionary I have consulted says, is quackery, then I fear that the term is of a wider application than many of us are prepared to accept, and some most reputable and esteemed members of our profession may be accused of an odious charge. They may be as wise as serpents and as harmless as doves, yet if they hiss or coo too loud . . .

The self-laudatory recommendations of JOHN TAYLOR certainly lay him open to the charge of quackery, for he had reduced this art almost to a science, and had his name been a more picturesque or a more euphonious one he would have added a new word to the English language.

John Taylor was an M.D. of half a dozen Universities that then ranked high in the schools of learning, and whose medical degrees were accepted in this country. He was the son of a doctor, and his son and grandson were doctors also. He studied at St. Thomas's under Cheselden, and was in practice in Norfolk as a surgeon before he forsook the narrow path and chose the broad one, which, wherever it may lead to, sometimes carries people to success in worldly goods and reputation in high quarters. Taylor made money. His house was crowded with persons seeking his advice on diseases of the eye, his speciality. He was appointed not only oculist to George III, but, according to his son's statement, to at least three other kings, several electors of the Holy Roman Empire, and to dozens of reigning princes. His methods of advertising were simple. He travelled about England and the Continent, set up his booth in the market-place, and if he had not a brass band to noise him abroad he was not forgetful of effect in other ways. He "appeared dressed in black, with a long light ty'd wig, ascended a scaffold behind a large table raised about two feet above the ground and covered with an old piece of tapestry, on which was laid a dark-coloured cafoy chariot seat, with four black bunches (used upon hearses) tyed to the corners for tassels, four large candles on each side of the cushion, and a quart decanter of drinking water, with half a pint glass to moisten his mouth." By an irony of fate Taylor died blind at Prague in 1767 in a convent. What he was doing there I do not know.

Johnson said of Taylor that he "was the most ignorant man I ever knew, but sprightly. . . . Taylor challenged me twice to talk Latin with him. I quoted some Horace, which he took to be part of my own speech. He said a few words well enough."

The other quack was WARD, whom Johnson described as the dullest man he had ever known. Ward's nickname was Spot, from a mark on his face. He is said to have been a very stout man, travelling about the country in an open coach drawn by four horses. To show how quacks of this type were favoured, I may mention that Ward was called in to cure George II of his headaches, and given an allowance from the king to treat the poor, with sumptuous rooms provided.

This, gentlemen, concludes my paper. I trust that I have been able to make the subject that I selected an interesting one to you. For my own part, anything connected with the history of our profession is instructive, and I may have been fortunate enough to have chosen a subject that is new to many of you.

[Since the above address was delivered my friend Dr. Humphry Rolleston has called my attention to an account of a *post-mortem* examination on the body of Dr. Johnson, published by Mr. James Squibb in the *London Medical Journal*, vol. i, p. 615. The account is from a MS. folio of "Dissections," by James Wilson,

Lecturer on Anatomy in the Hunterian School of Medicine, who did the autopsy on December 15th, 1784, for Mr. Cruikshank, in the presence of Drs. Heberden, Brocklesby Butler (probably Butter), and Mr. White. The evidence of the examination proves that Dr. Johnson died of hypertrophied heart following asthma, as shown by the emphysematous condition of the lungs and great size of the heart. The aortic valves were slightly ossified, a gall-stone was found in the gall-bladder, and the kidneys, more especially the left, had " hydatids," no doubt cysts. The pancreas is described as being remarkably enlarged, and the spleen to have " almost the feel of cartilage." The sarcocele appears to have been more " hydatids." The cranium was not opened].

